# Case Report: Triple-negative metaplastic breast carcinoma with primary resistance to neoadjuvant chemoimmunotherapy, refractory tumor necrosis-related fever, and rapid postoperative disease progression

**DOI:** 10.3389/fonc.2026.1943838

**Published:** 2026-09-17

**Authors:** LinHong Hu, YiFan Luo, Jing Luo

**Affiliations:** 1 School of Medicine, Southwest Medical University of China, Department of Breast Surgery The Affiliated Hospital of Southwest Medical University, Luzhou, China; 2 Department of Breast and Thyroid Surgery, The Affiliated Hospital of Panzhihua University, Panzhihua, China; 3 Department of Breast Surgery, Sichuan Provincial People's Hospital, University of Electronic Science and Technology of China, Chengdu, China

**Keywords:** case report, metaplastic breast carcinoma, neoadjuvant chemoimmunotherapy, primary resistance, tumor necrosis-related fever

## Abstract

**Background:**

Metaplastic breast carcinoma (MpBC) is a rare and highly heterogeneous breast malignancy. Although most cases exhibit a triple-negative phenotype, MpBC differs from conventional triple-negative breast cancer in its biological behavior, treatment response, and metastatic pattern. Neoadjuvant chemoimmunotherapy has become an important strategy for high-risk early-stage triple-negative breast cancer; however, the evidence supporting its use in MpBC remains limited.

**Case presentation:**

A 54-year-old woman was initially diagnosed with locally advanced triple-negative breast cancer of the left breast with ipsilateral axillary lymph node metastasis. A pembrolizumab-containing neoadjuvant regimen was initiated, with paclitaxel plus carboplatin as the initial chemotherapy backbone and planned sequential doxorubicin plus cyclophosphamide. After two cycles of initial neoadjuvant therapy, she developed recurrent high-grade fever, with extensive negative infectious workup and poor response to broad-spectrum antibiotics. Incision of the breast lesion revealed extensive gray-white necrotic tissue rather than typical purulent discharge. Multidisciplinary evaluation suggested tumor necrosis–related fever, and a modified radical mastectomy was performed. Fever resolved rapidly after surgery, and inflammatory markers decreased. Postoperative pathology confirmed high-grade triple-negative metaplastic breast carcinoma with Miller-Payne grade 1 response, a Ki-67 index of approximately 75%, and no definite axillary lymph node metastasis. Despite postoperative systemic treatment adjustment, the patient developed lumbar and hip symptoms with suspicious bone and soft-tissue lesions and rapidly progressed to cachexia. She died of systemic failure 5 months after surgery.

**Conclusion:**

This case highlights that persistent fever during neoadjuvant chemoimmunotherapy in MpBC should prompt careful exclusion of infection, while tumor necrosis–related fever and immune-inflammatory amplification should also be considered. In patients with suspected or confirmed MpBC who develop early tumor progression, necrosis, ulceration, fever, or deterioration in performance status, timely pathological review, reassessment of treatment response, and multidisciplinary decision-making are essential. Earlier surgery may be necessary to establish definitive pathology, control the local inflammatory source, and preserve subsequent treatment opportunities.

## Introduction

Breast cancer has become the most common malignancy among women worldwide, and metaplastic breast carcinoma (MpBC) represents a rare and highly heterogeneous subtype. Compared with conventional triple-negative breast cancer (TNBC), MpBC more commonly presents with a larger tumor size and locally advanced disease, with relatively less frequent axillary lymph node involvement but a higher risk of distant metastasis. In addition, its response to standard chemotherapy is generally poorer than that of conventional TNBC (1–4). Owing to its rarity and marked pathological heterogeneity, treatment for MpBC is still largely extrapolated from therapeutic strategies for TNBC.

With the KEYNOTE-522 trial establishing the clinical value of pembrolizumab combined with neoadjuvant chemotherapy in high-risk early-stage triple-negative breast cancer, chemoimmunotherapy has become an important treatment strategy in clinical practice (5). However, metaplastic breast carcinoma has been underrepresented in pivotal clinical trials. In real-world settings, some patients may derive benefit from immunotherapy, whereas others may develop primary resistance, rapid disease progression, or an abnormal inflammatory response after treatment (6). A prospective study with a small sample size has shown that neoadjuvant chemoimmunotherapy may increase the pathological complete response (pCR) rate in patients with MpBC; nevertheless, approximately 20% of patients still experienced disease progression during treatment and required early surgery (7). Therefore, when tumor enlargement, necrosis, fever, and rapid clinical deterioration occur during the treatment of MpBC, clinicians should consider a broad differential diagnosis, including infection, drug fever, immune-related adverse events, tumor necrosis-related fever, pseudoprogression, and hyperprogression.

This report describes a patient with high-grade triple-negative metaplastic breast carcinoma who developed refractory fever and extensive tumor necrosis during neoadjuvant chemoimmunotherapy, followed by rapid postoperative progression to cachexia and death. In conjunction with the existing literature, we discuss the potential mechanisms underlying the fever, the therapeutic implications of this case, and possible explanations for the rapid postoperative progression, with the aim of providing reference for the recognition and clinical decision-making of similar cases.

## Case presentation

### Patient information and initial treatment

The patient was a 54-year-old woman with no history of chronic disease, previous surgery, or drug allergy, and no personal history of malignancy or relevant family history. She presented to a local hospital because of a mass in the left breast. Core needle biopsy of the left breast mass and left axillary lymph node confirmed triple-negative breast cancer of the left breast with ipsilateral axillary lymph node metastasis (cT4bN1M0, stage IIIB). Neoadjuvant therapy was planned, consisting of paclitaxel plus carboplatin followed by doxorubicin plus cyclophosphamide, in combination with pembrolizumab. More than 20 days after the completion of the second cycle, she developed a recurrent fever, dry cough, nausea, and vomiting, with a maximum body temperature of 39 °C. Repeat breast magnetic resonance imaging suggested tumor progression accompanied by anemia. After symptomatic treatment, including anti-infective therapy, antiemetic treatment, and blood transfusion, her symptoms slightly improved, and she was subsequently admitted to our hospital. The major clinical events, diagnostic evaluations, therapeutic interventions, and outcomes are summarized in **Table 1**.

**Table 1:** Timeline of clinical events, diagnostic evaluation, and treatment interventions.

Time point	Clinical events and management	Key findings
Initial presentation	The patient presented with a left breast mass and ipsilateral axillary lymph node metastasis. Neoadjuvant therapy was initiated according to a high-risk TNBC regimen, consisting of paclitaxel plus carboplatin followed by doxorubicin plus cyclophosphamide combined with pembrolizumad.	Initial diagnosis: triple-negative breast cancer,cT4bN1M0(stage IIIB).
After completion of the second cycle of neoadjuvant therapy (approximately 20 days later)	The patient developed recurrent fever (maximum temperature 39°C), dry cough, nausea, and vomiting. Breast MRI showed enlargement of the primary lesion.	Suspected treatment resistance and disease progression.
Hospital admission	Comprehensive infectious evaluation and empirical anti-microbial therapy were performed.	Laboratory tests showed elevated WBC and CRP, while PCT remained low. microbiological investigations including blood/urine cultures and NGS were negative.
During hospitalization	Incision and drainage of the breast lesion were performed. Left modified radical mastectomy was performed after multidisciplinary discussion.	Extensive gray-white necrotic tissue without typical purulent secretion; pathology suggested tumor necrosis. After surgery,fever resolved rapidly after tumor removal.
Postoperative pathology	Histological examination of the surgical specimen was completed.	Diagnosis revised to high-grade triple-negative metaplastic breast carcinoma with sarcomatoid morphology. Tumor size was 7 cm, Miller-Payne grade 1, Ki-67 approximately 75%, and axillary lymph nodes were negative (0/19).
Approximately 1 month after surgery	Adjuvant systemic treatment was adjusted based on postoperative assessment and molecular testing.	PIK3CA mutation, TP53 mutation, NF1 frameshift mutation, EGFR amplification, and MSI-stable status were identified; capecitabine was followed by eribulin.
Approximately 5 months after surgery	The patient developed bone-related symptoms, cachexia and systemic deterioration.	Rapid clinical progression and death occurred;exact cause of death was unavailable.

### Clinical findings

The left nipple was retracted. In the lower inner quadrant of the left breast, the overlying skin was markedly erythematous and swollen, with visible superficial vessels; the skin involvement was less than one-third of the left breast. The central portion of the lesion was elevated, measuring approximately 5 cm × 6 cm, with skin ulceration, crust formation, and increased local skin temperature. A poorly defined mass measuring approximately 6 cm × 5 cm was palpable in the lower inner quadrant, with limited mobility. No other obvious abnormalities were noted on physical examination. Representative clinical and gross surgical findings are shown in **Figure 1**.

**Figure 1:**
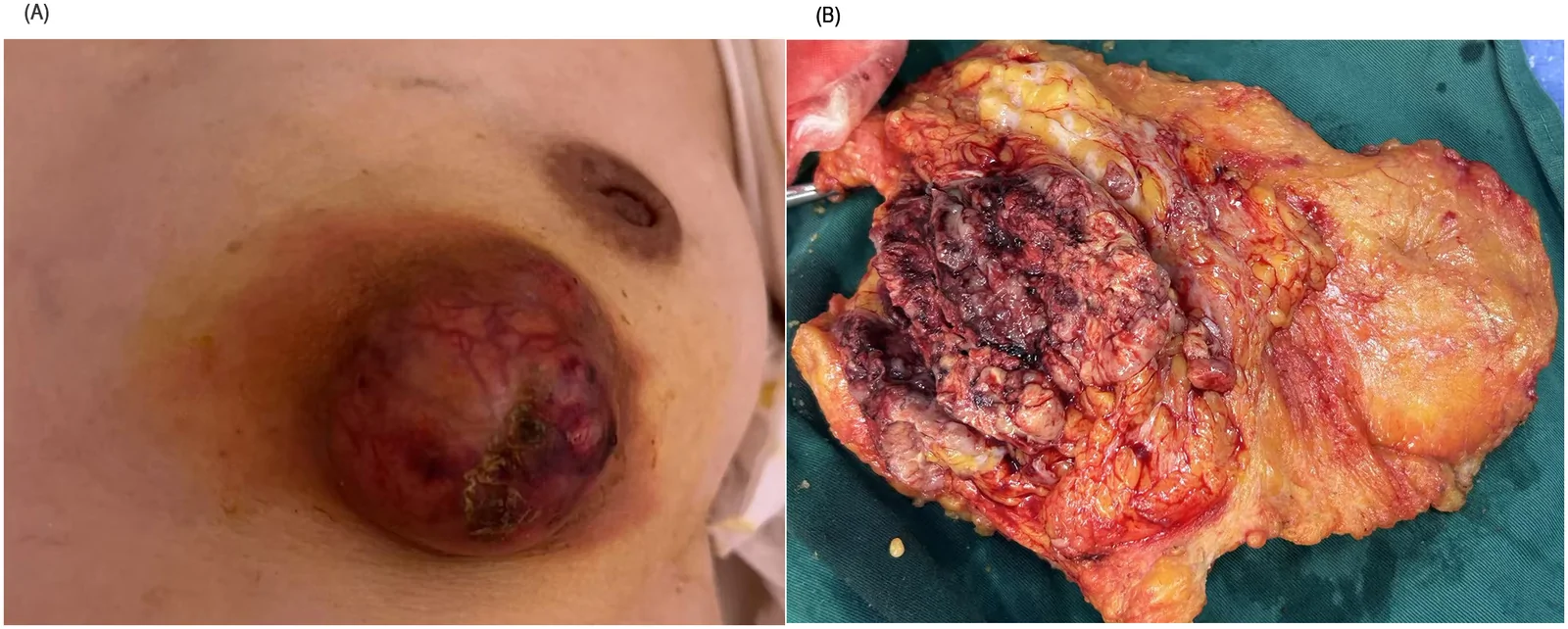
Local presentation of the left breast lesion and surgical specimen. Panel **(A)** shows the preoperative appearance of the left breast, with marked erythema and swelling of the skin, associated with ulceration, crust formation, and areas suggestive of tumor necrosis. Panel **(B)** shows the gross modified radical mastectomy specimen, demonstrating the resected breast tissue and a large tumor with extensive necrotic changes. The two panels illustrate the severe local presentation of the breast lesion before surgery and the corresponding gross appearance of the resected specimen.

### Diagnostic assessment

After admission, stat laboratory evaluation showed leukocytosis(WBC,21.13×10^9^/L),neutrophilia(79%),and an elevated C-reactive protein level(CRP,55.33mg/L),whereas the procalcitonin level remained low(<2ng/mL). Given that the patient was undergoing neoadjuvant therapy and presented with local erythema, ulceration, and increased skin temperature of the breast lesion, infectious fever was initially suspected. Empirical anti-infective treatment with moxifloxacin was therefore initiated, while a comprehensive etiological workup was performed. Tests for respiratory pathogens, serum (1→3)-β-D-glucan (G test) and galactomannan antigen (GM test), tuberculosis-specific testing, chest and abdominal CT scans, and repeated blood and urine cultures revealed no abnormalities. Metagenomic next-generation sequencing (mNGS) of both peripheral blood and tumor tissue was also negative.

Subsequently, incision of the lesion were performed. A large amount of gray-white necrotic material was observed locally, but no typical purulent discharge was identified. Pathological examination showed diffuse proliferation of atypical epithelioid cells accompanied by extensive degenerative necrosis, and the corresponding microbiological tests remained negative. Despite escalation of anti-infective therapy to broad-spectrum antibiotics, including piperacillin and meropenem, the patient continued to have a fever, persistently elevated inflammatory markers, and no obvious clinical improvement.

### Therapeutic intervention

Given the discordance between markedly elevated inflammatory markers and the absence of definite microbiological evidence, along with a lack of response to antibiotics and extensive tumor necrosis observed in the local lesion, a noninfectious inflammatory response secondary to tumor necrosis was highly suspected as the source of fever. After multidisciplinary discussion and exclusion of surgical contraindications, surgical treatment was recommended. The patient subsequently underwent modified radical mastectomy for left breast cancer. After surgery, her body temperature rapidly returned to normal, with no further persistent fever, CRP decreased from 73.69 mg/L preoperatively to 27.59 mg/L on postoperative day 1, while WBC decreased from 15.25 × 10^9^/L to 11.75 × 10^9^/L and subsequently normalized during follow-up.

### Follow-up and outcomes

Postoperative histopathological examination confirmed high-grade sarcomatoid carcinoma, a histological subtype of metaplastic breast carcinoma, of the left breast. The maximum tumor diameter was 7 cm. The response to neoadjuvant therapy was classified as grade 1 according to the Miller-Payne grading system. Immunohistochemically, the tumor was positive for CK, CAM5.2, and TRPS1 and negative for ER and PR, with HER2 scored as 0. The Ki-67 proliferation index was approximately 75%, and p53 staining was observed in approximately 65% of tumor cells. p63, CK5/6, SOX10, desmin, MyoD1, calponin, and synaptophysin were negative. No definite metastatic carcinoma was identified in the left axillary lymph nodes (0/19).

After postoperative wound healing, and based on the patient’s clinical condition and postoperative pathological findings, intensified adjuvant therapy with capecitabine was initially administered. Approximately 1 month after surgery, genetic testing of tumor tissue and peripheral blood revealed alterations, including PIK3CA mutation, TP53 mutation, NF1 frameshift mutation, and EGFR amplification; peripheral blood testing showed microsatellite stability. No molecular alteration was identified that could directly guide standard targeted therapy in this case. Given the large residual tumor burden, Miller-Payne grade 1 response, clinical progression during the preceding taxane-, platinum-, and pembrolizumab-containing neoadjuvant therapy, and the perceived high risk of early systemic progression, the treatment strategy was reassessed by the multidisciplinary team. Capecitabine was subsequently discontinued and systemic treatment was switched to eribulin as an individualized postoperative treatment strategy.

However, approximately 2 months after surgery, the patient developed right hip pain accompanied by weight-bearing weakness. Magnetic resonance imaging (MRI) of the spine revealed abnormal signals involving the L1 and L3 vertebral bodies and the posterior elements of L3, with associated paravertebral soft-tissue swelling and spinal canal stenosis causing nerve root compression. Pelvic and hip MRI also demonstrated suspicious lesions in the right ischial tuberosity, ilium, and proximal left femur, accompanied by edema of the right gemellus inferior and quadratus femoris muscles. Although these MRI findings raised concern for metastatic involvement, whole-body bone scan and SPECT failed to identify definite metastatic lesions. Representative MRI findings of the right acetabulum and ischial tuberosity are shown in **Figure 2**. Following multidisciplinary evaluation by orthopedic, medical oncology, and radiation oncology teams, palliative radiotherapy was delivered to the L3 vertebra and right hip joint at a dose of 30 Gy in 10 fractions. Denosumab was also added, but her symptoms did not improve significantly. Her condition subsequently progressed, with the development of cachexia, and she died at an outside hospital approximately 5 months after surgery. The exact cause of death remains unknown.

**Figure 2:**
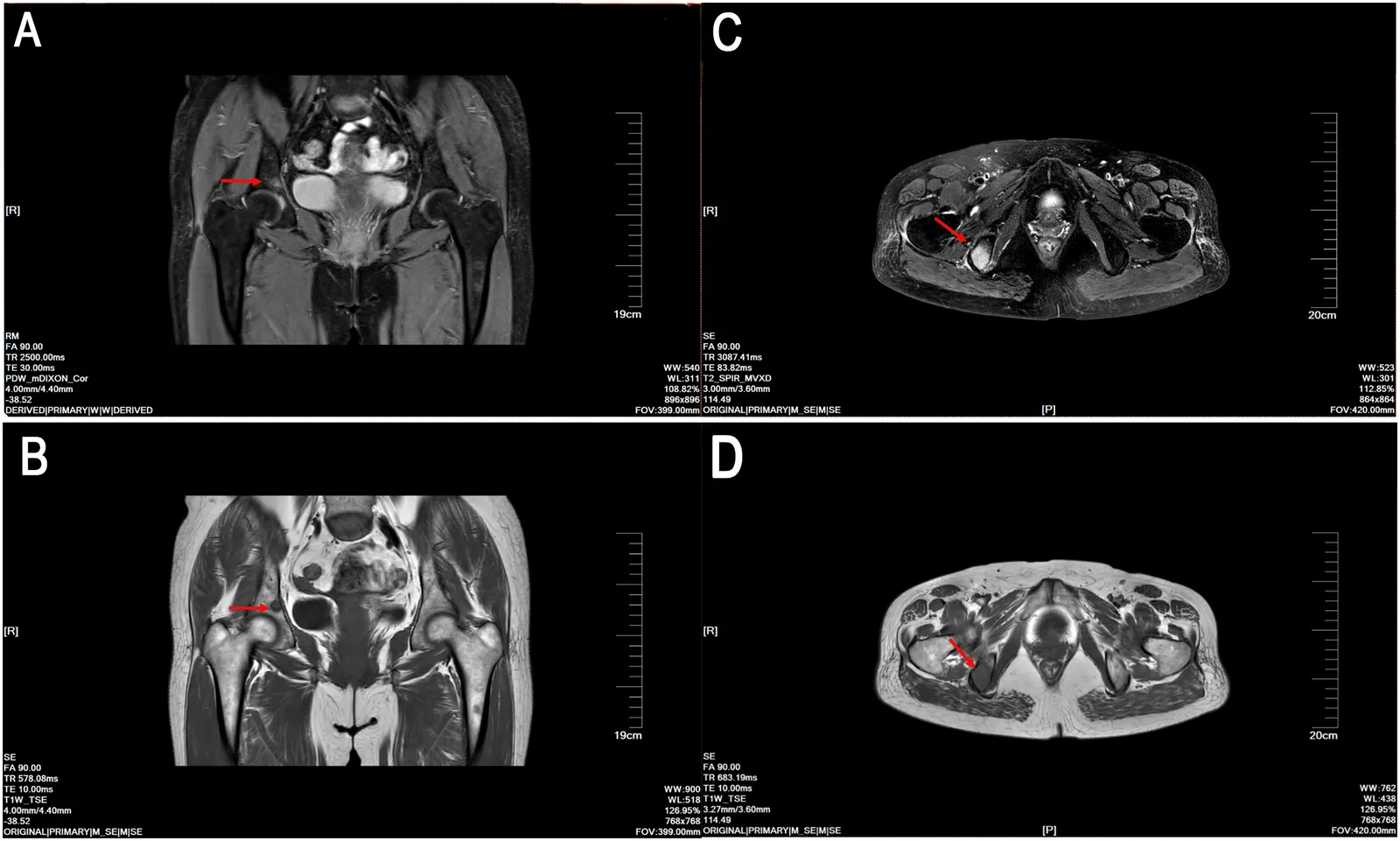
Representative magnetic resonance imaging findings of the right hip and ischial tuberosity. Panels **(A)** and **(B)** show magnetic resonance images of the right acetabular region, with red arrows indicating abnormal osseous changes involving the right acetabulum. Panel **(A)** is a T2-weighted image, whereas Panel **(B)** is the corresponding T1-weighted image. Panels **(C)** and **(D)** show the right ischial tuberosity, with red arrows indicating abnormal signal intensity in this region. Panel **(C)** is T2-weighted and Panel **(D)** is T1-weighted. These findings were clinically suspicious for metastatic involvement but were not histologically confirmed.

## Discussion

This case also highlights that increased local skin temperature and erythema should not be equated with infection, nor should tumor necrosis necessarily be interpreted as a sign of effective treatment. In patients with cancer, especially those who develop fever after chemotherapy, infectious fever must first be excluded. In the present case, the patient had clinical signs suggestive of infection, including local erythema, ulceration, and increased skin temperature. However, persistently low procalcitonin levels, repeatedly negative microbiological tests, absence of pathogens on metagenomic next-generation sequencing, lack of typical purulent discharge after incision, pathological evidence of tumor cells with extensive degenerative necrosis, failure of escalated anti-infective therapy, and rapid defervescence after surgery all supported the diagnosis of tumor necrosis–related fever.

Tumor fever is usually a diagnosis of exclusion. Its underlying mechanisms are thought to involve tumor necrosis, immune-cell infiltration, and the release of endogenous pyrogenic mediators such as interleukin-1, interleukin-6, and tumor necrosis factor-α (8, 9). The naproxen test, as reported in previous studies, has often been used as an adjunctive strategy to help distinguish tumor fever from infectious fever, but it cannot replace infectious workup or local lesion assessment (8, 9). In this case, the naproxen test and cytokine profiling were not performed, which represents one of the limitations of this report. Nevertheless, the resolution of fever after resection of the necrotic tumor provided an important diagnostic clue.

MpBC is often characterized by more pronounced epithelial–mesenchymal transition, abundant stromal components, tumor stem cell-like features, and aberrations in multiple molecular pathways, all of which have been associated with chemoresistance and enhanced invasiveness (1, 3, 4, 10). In the present case, the postoperative Miller-Payne grade was 1, indicating no substantial reduction in overall tumor cellularity after neoadjuvant therapy. In addition, the maximum tumor diameter reached 7 cm, the Ki-67 index was approximately 75%, and extensive necrosis was present. These findings are more consistent with rapid tumor proliferation leading to relative insufficiency of blood supply and necrosis-associated inflammatory release, rather than a favorable pathological response to treatment.

The initial core needle biopsy did not identify a definite metaplastic component, whereas pathological re-review of the surgical specimen confirmed sarcomatoid carcinoma, a subtype of metaplastic breast carcinoma. This also reflects the marked heterogeneity of this tumor type and the inherent limitations of core biopsy sampling. Subsequent genetic testing of tumor tissue revealed multiple pathway-related alterations together with microsatellite stability, suggesting that the benefit from immunotherapy in this case may have been limited and that the tumor had features associated with treatment resistance. Therefore, for patients with breast cancer who develop clinical progression or unusual inflammatory manifestations during treatment, early pathological consultation, repeat biopsy, or careful review of the surgical specimen is of substantial clinical value.

The optimal treatment sequence for MpBC remains unsettled. A 2025 NCDB analysis published in *Annals of Surgical Oncology*, which included 5,136 patients with stage I–III MpBC, reported significantly better 5-year overall survival in patients who underwent upfront surgery followed by adjuvant therapy than in those who received neoadjuvant chemotherapy followed by surgery; the pCR rate after neoadjuvant therapy was only 4.92% (11). These findings suggest that the benefit of conventional neoadjuvant chemotherapy may be limited in MpBC and that some resectable patients may be better served by early surgical treatment. With the introduction of immune checkpoint inhibitors into the treatment of high-risk TNBC (5), however, new neoadjuvant strategies for MpBC have also emerged. In a small prospective study from Memorial Sloan Kettering, 15 patients with early-stage MpBC received KEYNOTE-522–like neoadjuvant chemoimmunotherapy. Although some patients achieved pCR, 20% experienced disease progression during treatment, requiring discontinuation of neoadjuvant therapy and early surgery (7). Nevertheless, for patients with highly suspected or confirmed MpBC, the development of tumor enlargement, necrosis, ulceration, persistent fever, or deterioration in performance status during the early phase of neoadjuvant therapy should be recognized as potential signs of lack of benefit. In such situations, clinicians should promptly shift the treatment strategy, reassess the disease, and when necessary, adjust the treatment sequence through multidisciplinary discussion so as not to miss the window for local control and definitive pathological evaluation.

The significance of immunotherapy in this case should also be interpreted with caution. MpBC may show high PD-L1 expression and abundant FOXP3-positive tumor-infiltrating lymphocytes, and responses to PD-1/PD-L1–directed therapy have been reported in some patients with metastatic MpBC (2, 10, 12), providing a biological rationale for immunotherapy. However, postoperative molecular testing in this patient showed microsatellite stability, suggesting a potentially limited benefit from PD-1/PD-L1 blockade. In addition, alterations involving PIK3CA, TP53, NF1, and EGFR amplification were detected, indicating aberrations in multiple molecular pathways (13), which may help explain the poor response to neoadjuvant therapy in this case. Together with a Miller-Payne grade 1 response, the overall findings are more consistent with primary resistance.

It should be noted that immune checkpoint inhibitor therapy may also be associated with pseudoprogression, hyperprogression, or excessive tumor-associated inflammation. In previously reported cases of renal cell carcinoma, some patients developed radiological progression and rapid clinical deterioration after immunotherapy; however, biopsy of the progressing lesions revealed extensive necrosis and lymphohistiocytic infiltration without definite viable tumor cells, suggesting that an excessive antitumor inflammatory response itself may contribute to adverse clinical outcomes (14). In addition, immune checkpoint inhibitors can induce cytokine release syndrome or hemophagocytic lymphohistiocytosis, which may manifest as fever, elevated inflammatory markers, hypotension, and organ dysfunction, with onset ranging from immediately after infusion to several weeks after treatment (15).The present case shares several features with these phenomena, including fever, tumor necrosis, and systemic inflammation occurring in the context of chemoimmunotherapy, as well as rapid defervescence after resection of the primary lesion, suggesting that the tumor itself may have been an important source of inflammatory burden. Nevertheless, the postoperative pathological specimen still showed a large amount of residual viable tumor, which cannot be explained by typical pseudoprogression or immune-cell infiltration alone. Moreover, because data on tumor growth kinetics before and after treatment, immune-cell infiltration profiles, and serum cytokine levels were unavailable, immune-related hyperprogression or cytokine release syndrome could not be confirmed. Overall, this case is more suggestive of primary resistance and progression of MpBC, with tumor necrosis and immune-mediated inflammation jointly contributing to refractory fever and systemic inflammatory burden.

This case also suggests that systemic treatment for MpBC should not simply rely on extrapolation from conventional TNBC regimens. Genetic testing in this patient indicated possible aberrations in the PI3K/AKT/mTOR, RAS/MAPK, and EGFR-related pathways 13. Although these findings do not point to a specific standard targeted therapy, they may help explain the resistance to neoadjuvant treatment in this case and may also provide clues for subsequent clinical trial screening. Immune-based combinations, pathway-directed strategies, anti-angiogenic approaches, and antibody-drug conjugates remain important investigational directions for MpBC, but evidence specific to this rare subtype remains limited (12).

Rapid postoperative death represents another distinctive feature of this case. Superficially, the absence of axillary lymph node metastases after surgery (0/19) and complete resection of the primary lesion might suggest adequate local disease control. However, the metastatic pattern of MpBC often differs from that of conventional breast cancer, with relatively less prominent axillary lymph node involvement but a higher propensity for hematogenous dissemination and distant metastasis (1, 3). In the present case, the large primary tumor, skin involvement, high Ki-67 index, Miller-Payne grade 1 response, and the early postoperative appearance of abnormal signals in the pelvis, lumbar vertebrae, and paravertebral soft tissue all indicated an extremely high risk of systemic residual disease. A negative whole-body bone scan could not completely exclude early bone marrow infiltration, osteolytic lesions, or paravertebral soft-tissue metastasis, because bone scintigraphy primarily reflects osteoblastic activity, whereas MRI is more sensitive for detecting bone marrow signal abnormalities and soft-tissue involvement. In diagnostically challenging situations, PET-CT or biopsy of the suspicious lesion may provide more definitive characterization (16). As the patient did not undergo biopsy of the suspicious lesions or autopsy, the cause of death cannot be simply attributed to bone metastasis or immune-related events. Nevertheless, given the rapid clinical deterioration, worsening pain, and decline in performance status, occult distant progression was highly suspected to have contributed to her early postoperative death.

Cachexia is not merely the result of inadequate nutritional intake but a systemic syndrome jointly driven by the tumor, host immune inflammation, and metabolic dysregulation. Inflammatory mediators such as interleukin-6, tumor necrosis factor-α, and interleukin-1β can promote muscle protein breakdown, lipid mobilization, anorexia, acute-phase responses, and increased energy expenditure (17, 18). In the present case, the patient had persistent fever, elevated WBC count and CRP level, and extensive tumor necrosis before surgery, indicating a marked systemic inflammatory state. Although surgery removed the major local source of necrosis-associated fever, if occult micrometastatic disease had already been present preoperatively or if the systemic inflammation–metabolism axis had already been activated, irreversible cachexia could still have developed within a short period after surgery. For similar patients, postoperative follow-up should not focus solely on local wound healing and axillary lymph node status. Close monitoring of body weight, appetite, serum albumin, CRP, hemoglobin, pain, muscle strength, and imaging abnormalities is also essential. Once bone pain, weight-bearing weakness, or rapid weight loss occurs, early systemic imaging reassessment and supportive care should be initiated.

The strength of this case lies in its well-documented clinical course. The temporal relationship among the diagnostic workup before and after the onset of fever, the lack of response to anti-infective therapy, evidence of extensive local tumor necrosis, resolution of fever following surgical intervention, and the subsequent clinical outcome provides valuable insight into the recognition of tumor necrosis–related fever in clinical practice. Nevertheless, this case has several important limitations. Neoadjuvant therapy was initiated at an outside institution, and baseline and on-treatment imaging data were incomplete, precluding accurate assessment of tumor growth kinetics. In addition, the naproxen test, serum cytokine profiling, PD-L1 combined positive score (CPS), tumor-infiltrating lymphocyte (TIL) evaluation, and multiplex immunohistochemical analyses were not performed, limiting our ability to distinguish tumor necrosis–related fever from excessive immune activation associated with immunotherapy. Furthermore, the postoperative lumbar spine and pelvic lesions were not histologically confirmed, and the exact cause of death could not be established because an autopsy was not performed. Given these limitations, the conclusions drawn from this report should be interpreted as providing clinical insight and a diagnostic framework for similar cases, rather than definitive evidence to support a particular treatment sequence.

## Conclusion

This case describes a patient with MpBC who exhibited primary resistance to neoadjuvant chemoimmunotherapy, developed refractory tumor fever, and experienced rapid postoperative death. It suggests that, when a persistent fever occurs during treatment in patients with MpBC, clinicians should carefully consider the possibility of tumor necrosis-related fever and amplified immune-inflammatory responses after adequately excluding infection. If fever is accompanied by tumor progression, necrosis with ulceration, or deterioration in performance status, timely pathological review, reassessment of treatment response, and multidisciplinary decision-making are warranted. When necessary, earlier surgery should be considered to establish a definitive pathological diagnosis, control the local inflammatory source, and preserve opportunities for subsequent treatment. Even in the presence of negative axillary lymph nodes after surgery, high-intensity follow-up and early supportive intervention should be guided by the residual burden of the primary tumor, pathological treatment response, and systemic inflammatory status.

## Data Availability

The original contributions presented in the study are included in the article/supplementary material. Further inquiries can be directed to the corresponding author.
